# Mortality and morbidity among people living close to incinerators: a cohort study based on dispersion modeling for exposure assessment

**DOI:** 10.1186/1476-069X-10-22

**Published:** 2011-03-24

**Authors:** Andrea Ranzi, Valeria Fano, Laura Erspamer, Paolo Lauriola, Carlo A Perucci, Francesco Forastiere

**Affiliations:** 1 Environmental Health Reference Centre, Regional Agency for Environmental Prevention of Emilia Romagna, Modena, Italy; 2 Department of Epidemiology - Lazio Regional Health Service, Rome, Italy

## Abstract

**Background:**

Several studies have been conducted on the possible health effects for people living close to incinerators and well-conducted reviews are available. Nevertheless, several uncertainties limit the overall interpretation of the findings. We evaluated the health effects of emissions from two incinerators in a pilot cohort study.

**Methods:**

The study area was defined as the 3.5 km radius around two incinerators located near Forlì (Italy). People who were residents in 1/1/1990, or subsequently became residents up to 31/12/2003, were enrolled in a longitudinal study (31,347 individuals). All the addresses were geocoded. Follow-up continued until 31/12/2003 by linking the mortality register, cancer registry and hospital admissions databases. Atmospheric Dispersion Model System (ADMS) software was used for exposure assessment; modelled concentration maps of heavy metals (annual average) were considered the indicators of exposure to atmospheric pollution from the incinerators, while concentration maps of nitrogen dioxide (NO_2_) were considered for exposure to other pollution sources. Age and area-based socioeconomic status adjusted rate ratios and 95% Confidence Intervals were estimated with Poisson regression, using the lowest exposure category to heavy metals as reference.

**Results:**

The mortality and morbidity experience of the whole cohort did not differ from the regional population. In the internal analysis, no association between pollution exposure from the incinerators and all-cause and cause-specific mortality outcomes was observed in men, with the exception of colon cancer. Exposure to the incinerators was associated with cancer mortality among women, in particular for all cancer sites (RR for the highest exposure level = 1.47, 95% CI: 1.09, 1.99), stomach, colon, liver and breast cancer. No clear trend was detected for cancer incidence. No association was found for hospitalizations related to major diseases. NO_2 _levels, as a proxy from other pollution sources (traffic in particular), did not exert an important confounding role.

**Conclusions:**

No increased risk of mortality and morbidity was found in the entire area. The internal analysis of the cohort based on dispersion modeling found excesses of mortality for some cancer types in the highest exposure categories, especially in women. The interpretation of the findings is limited given the pilot nature of the study.

## Background

Several studies on the possible health effects related to residing close to incinerators have been published and well-conducted reviews are available [1-3]. Results from ecological studies have suggested associations with some reproductive outcomes (infant deaths and congenital malformations [4]; birth defects [5]; congenital anomalies and stillbirths [6]; gestational age [7]) and with cancer (all cancer, larynx, lungs, esophagus, stomach, intestine, liver, kidneys, bladder and breast) [8,9]. However, there are several weaknesses of these results due to design issues such as lack of exposure information and use of surrogate measures such as distance from the source, lack of control for potential confounders. Therefore several uncertainties limit the overall interpretation of the findings. More detailed studies on incinerators in France and in Italy suggest an increased risk for non-Hodgkin's lymphoma [10-12], soft-tissue sarcoma [13,14] and urinary tract birth defects [15].

These contradictory results do not permit resolution of the issue, and concerns of people living in areas near incinerators require more in-depth studies [1-3]. Recent investigations have used dispersion models to assess population exposure [11,12], an approach that provides a better exposure assessment than studies based on distance from the source. However, such studies have used health data at the aggregate level with numerator and denominator information coming from different sources and with limited possibility of adjusting for confounding related to socioeconomic status. The present work proposes an approach in which exposure assessment is based on geographical characterization by means of dispersion models, outcome information is collected within an individual-based retrospective longitudinal study, and an area-based socioeconomic status index is considered in the analysis.

The aim of the study was to evaluate the health effects of emissions from two incineration plants in the nearby resident population (Emilia-Romagna, Italy), while considering the effects of other environmental stressors and socioeconomic status. Given the limited size of the study and the consequent low statistical power, we considered this approach as a pilot study before addressing the issue in the future using the same methodology to evaluate the effects of other seven plants located in the Emilia-Romagna region.

## Methods

### Characteristics of the plants

**Figure 1:**
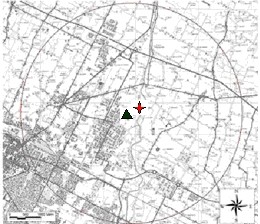
**Study area around incinerators: black triangle indicates Municipal Solid Waste Incinerator, red star indicates Hospital Waste Incinerator**.

The municipality of Forlì (107,827 inhabitants in 2001) is located in the Po valley (Emilia Romagna region, Northern Italy). Two incinerators are located about 3 km from the town, about 200 m from each other (Figure 1):

• an incinerator of municipal solid waste (MSW) that began operating in 1976 with two lines (capacity of 35,000 Nm^3^/h, stack height 60 m.); a first renewal was completed in 1993 (capacity of 58,000 Nm^3^/h), and, finally, in 2000 a plant renovation brought the total capacity to 60,000 Nm^3^/h (stack diameter 2.2 m, exit velocity 6.5 m/s).

• an incinerator for hospital waste (HW) that began activity in 1991 (stack height 39 m., capacity 7,500 Nm^3^/h), the authorized capacity was extended to 9, 500 Nm^3^/h in 1997 and to 12,500 Nm^3^/h in 1999. In 2003, a new plant was activated with a total capacity of 21,500 Nm^3^/h and a stack of 49 m of height (stack diameter 0.95 m, exit velocity 10 m/s).

The position and the structure of the two plants has remained substantially the same over time (the only substantial change was the height of the HW chimney). Historical emissions data based on routine checks are only available back to 1994 and indicate that between 1994 and 1996 high values of dioxins were emitted by the MSW plant [16]. The emissions of main pollutants significantly decreased in recent years, as shown in the Table 1 for the MSW plant.

**Table 1:** MSW plant emissions of Total Suspended Particulates (TSP), Mercury (Hg), Cadmium (Cd), and Dioxins (PCDD+PCDF) in 1994/1996 and in 2008.

Period	Unit of measurement	1994-1996	2008	ratio
TSP	mg/Nm^3^	4.6	0.991	0.214
Hg	μg/Nm^3^	23.2	0.476	0.020
Cd*	μg/Nm^3^	21.0	0.422	0.020
PCDD+PCDF**	ng/Nm^3^	128.7	0.018	0.0001

* Cd only in 1994-96, Cd+Tl in 2008

** Total dioxins in 1994-96, TEQ dioxins in 2008

### The study area

The study area was defined as the 3.5 km radius circle around the two incinerators (the central point was the middle distance between the two) (Figure 1), on the basis of the previous literature [8,10] and the results of the dispersion model (see below). Most of the area included in the study is used for agriculture; the remaining territory and the borders of the urban area are occupied by three small industrial areas and by an urban district of the city of Forlì. Besides the incineration plants, the other main sources of air pollution are traffic (from urban area and two major roadways), and domestic heating during winter.

### ADMS simulations

To define the exposure conditions in the area, we used the results of an environmental study conducted during the period 1997-2000 [17]. On the basis of emission inventories of all the environmental factors currently present in the study area (road traffic, industrial plants, incinerators and heating), the quasi-Gaussian model Atmospheric Dispersion Modeling System (ADMS) Urban 2.2 [18] was used to simulate the impact of the different emission sources. Estimated annual average concentration maps of several pollutants (NO_2_, SOx, COV, CO, TSP, C6H6, HCl and heavy metals) were produced. Heavy metals were the entire set included in the current legislation for industrial emissions, i.e.: lead, cadmium, mercury, antimony, arsenic, chromium, cobalt, copper, manganese, nickel, vanadium, tin.

Based on these preliminary models, points of maximum and minimum fallout for pollutants emitted from the incinerators were identified. Moreover, a point of maximum fallout for all sources was identified. Several monitoring campaigns were conducted to measure concentrations at the points of maximum and minimum fallout using passive samplers, bulk passive sampler, Wet&Dry and soil depositions for measurements and determinations of SOx, NO_2_, CO, TSP, BTX, HCl, PCDD/F, PAH, PCB and heavy metals. The best results were found for heavy metals since the highest concentrations were found at the point of maximum fallout from incinerators, the lowest concentrations at the point of minimum fallout, while intermediate values were found at the point of maximum fallout for all sources. The same was not true for the other measured pollutant, including dioxins and hydrochloric acid, with no clear relation between the estimated points of minimum and maximum fallout and the actual concentrations.

As a result, we decided to consider heavy metals as the tracer of pollution from incinerators. We ran the ADMS model using the characteristics of the plants, and authorized limits of emissions for heavy metals as for early 1990s. We are aware that the regulations changed during the following ten years but we preferred to represent exposure as it was in the past. The current situation was also simulated using more recent authorized limit of emissions (year 2005), in order to verify the exposure gradients in the different scenarios.

Nitrogen dioxide (NO_2_) was identified as the best tracer of air pollution from all other sources. However, it was not possible to reconstruct the emission scenario for the other environmental pressure factors in the past, so current emission factors were used based on the available emission inventories and the authorized values for emissions.

The ADMS requires hourly information about wind speed/direction, total cloud amount, and air temperature to calculate atmospheric boundary layer parameters. Hourly surface meteorological data from the Meteorological Service of the Regional Agency for Environmental Prevention (ARPA Emilia Romagna) station network were acquired to build the meteorological file for the years of simulation.

Model outputs were mapped using ArcView GIS 8.2 [19] on an "intelligent" grid based on 100 × 100 fixed nodes (rectangle of 7518 × 7618 m.) of the study area to develop concentration grids for each tracer pollutant. We constructed map layers by means of surface interpolations (simple kriging) and obtained the concentration maps for heavy metals and NO_2._

### Enrolment of the cohort and follow-up procedures

The General Registry Office of Forlì was the data source for the enrolment of the cohort. Subjects who resided in the study area on 1/1/1990, or who subsequently became residents until 31/12/2003, were enrolled in a retrospective longitudinal study. For those subjects who entered in the area after 1/1/1990, a minimum of five years of residence was required before starting the follow- up. The follow-up was carried out through record linkage with the regional mortality database (from 1990 to 2003) which includes all deaths of the resident population from anywhere in the country. The Cancer Registry database (from 1990 to 2003) and the Hospital Admissions database (from 1999 to 2003), provided by the Romagna Cancer Registry and the Regional Health Information System, were also used. Hospital admissions for specific causes were considered relevant for the study to evaluate cardiovascular and respiratory morbidity; the first admission for each subject and each cause was included in the analysis. Subjects were considered at risk for the various outcomes (mortality, cancer incidence, or hospitalization) until they died, moved outside the region, or until the last day of the follow-up (31/12/2003). The General Registry Office traced the geographical coordinates of all residences after 1990 but residential histories before 1990 were not available. The relevant residence of each subject was his or her home in 1990 or the first residence for later arrivals.

### Exposure indicators

We used the geo-coded addresses to define residential exposures. Each subject in the cohort was assigned a value of heavy metals and NO_2 _corresponding to the estimated map values at the residence from the ADMS dispersion model. Quartiles of the distribution of the total population were calculated for heavy metals and NO_2_. The final categorization, however, was finely adjusted to consider the natural cut-off points.

Based on census data from 2001 for the area of Forlì, an area-based indicator (census block) of socio-economic status (SES) was defined. A total of 1,116 census areas with at least 20 people for the town of Forlì were considered. Four census variables were chosen to represent different levels of social advantage: education, employment, housing conditions, and family composition. A factorial analysis was conducted, to define a composite indicator of socioeconomic position, combining algebraic indicators and using the weight factor scores. The final categories of the SES indicator (from low to high social class) were based on the population distribution (quintiles) of the composite index.

### Data analyses

For all health outcomes considered, a preliminary analysis was carried out in order to compare mortality and morbidity of the entire cohort with an external reference area. SMR (Standardized Mortality Ratio) and SIR (Standardize Incidence Ratio) were calculated to compare mortality and cancer incidence of the cohort with the regional population or with the local health district population for hospital admissions.

In the integrated database of the cohort, for each individual we had demographic information, SES level, exposure levels at the residence for heavy metals and NO_2_, date of entry, date of exit and several outcomes (mortality, incidence, and hospitalization). Person-years at risk were calculated by calendar period, gender and age. Age and socioeconomic status adjusted rate ratios (RR) and 95% Confidence Intervals (95% CI) of the association with heavy metal exposure were estimated with Poisson regression separately for males and females and for all the health outcomes considered, using the lowest exposure category as the reference. The choice of the relevant outcomes was based on the literature review. Analyses for soft tissue sarcoma were performed also by combining the results for men and women. To evaluate the possibility of confounding from traffic-related air pollution, we ran an additional analysis using NO_2 _levels as an adjustment factor. We considered *a priori *that the associations to be noted were those with an increasing trend in the adjusted rate ratios across the exposure categories and/or a statistically significant rate ratio in the third or fourth quartiles when compared with the first one.

The statistical package STATA was used for all analyses [20].

## Results

**Figure 2:**
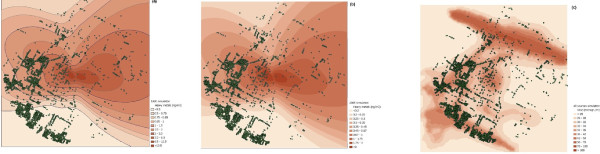
**ADMS concentration map for incinerators in different periods (a) (b) and other sources (c)**.

The estimated annual average heavy metals concentration throughout the study area was 1.08 ng/m^3 ^(standard deviation, SD = 1.03) in 1990 and 0.46 ng/m^3 ^(SD = 0.45) in 2005. The annual average NO_2 _value in 2005 was 37.46 μg/m^3 ^(SD = 6.56). The concentration maps for heavy metals estimated for 1990 and for 2005 are illustrated in Figure 2a and Figure 2b, respectively. The relative population distributions are rather similar in the two exposure scenarios, although the absolute values are different. Figure 2c illustrates the NO_2 _concentration map in 2005 indicating no spatial correlation with predicted heavy metals concentrations.

A total of 31,347 individuals were enrolled in the cohort (51.7% females). Their main characteristics in relation to the four categories of residential exposure to heavy metals are described in Table 2. The gender and age distribution was rather similar over the four exposure categories (although people in the second category tended to be younger than in other categories) whereas socioeconomic status and NO_2 _exposure were different. People in the highest heavy metal exposure categories tended to have a lower socioeconomic status than those in the lowest categories (low social class: 8.6% in the lowest heavy metal category versus 20.6% in the highest) while NO_2 _levels were highest in the low heavy metal group. As indicated at the bottom of the table, most of the cohort members (93.1%) remained in the initial heavy metals category throughout the follow-up. A total of 3,407 deaths (1,753 males, 1,654 females) were observed during the study period with a total of 354,702 years of observation.

**Table 2:** Characteristics of the cohort of residents in the area of Coriano (Italy) during the period 1990-2003 by estimated air concentration of healvy metals.

	Heavy metals air concentration									
	I		II		III		IV			
	<0.5 ng/m3		0.5 -1 ng/m3		1-2 ng/m3		>2 ng/m3		Total	
	n	%	n	%	n	%	n	%	n	%
										
**Total**	10391	33.1	7961	25.4	9400	30.0	3595	11.5	31347	100.0
										
**gender**										
**male**	4966	47.8	3775	47.4	4602	49.0	1811	50.4	15154	48.3
**female**	5425	52.2	4186	52.6	4798	51.0	1784	49.6	16193	51.7
										
**age (years)**										
**0 -44**	5051	48.6	4178	52.5	4414	47.0	1720	47.8	15363	49.0
**45 -59**	2457	23.6	1808	22.7	2347	25.0	894	24.9	7506	23.9
**60-74**	1780	17.1	1266	15.9	1548	16.5	614	17.1	5208	16.6
**75+**	1103	10.6	709	8.9	1091	11.6	367	10.2	3270	10.4
										
**Socioeconomic status**										
**low**	871	8.6	405	5.3	1294	14.7	656	20.6	3226	10.8
**medium low**	2289	22.5	659	8.6	3844	43.7	1088	34.1	7880	26.4
**medium**	2893	28.4	1633	21.3	2650	30.1	942	29.6	8118	27.2
**medium high**	3311	32.6	2515	32.8	932	10.6	500	15.7	7258	24.3
**high**	805	7.9	2447	31.9	74	0.8	0	0.0	3326	11.2
										
***missing***	*222*	2.1	*302*	3.8	*606*	6.4	*409*	*11.4*	*1539*	*4.9*
										
**NO**_**2 **_**concentration**										
**<30 μg/m**^**3**^	2624	25.3	1404	17.6	2851	30.3	956	26.6	7835	25.0
**31-35 μg/m**^**3**^	2369	22.8	4188	52.6	3554	37.8	1478	41.1	11589	37.0
**36-40 μg/m**^**3**^	2012	19.4	2012	25.3	1896	20.2	932	25.9	6852	21.9
**>40 μg/m**^**3**^	3386	32.6	357	4.5	1099	11.7	215	6.0	5057	16.1
										
**Change of exposure during follow-up**										
**never**	9744	93.8	7175	90.1	8737	92.9	3252	90.5	29185	93.1
**one o more changes**	647	6.2	786	9.9	663	7.1	343	9.5	2462	7.9
										
**Person years**	120568		90980		103015		40139		354702	

Totals for different variables may vary because of missing values

The analyses of the overall cohort mortality compared with regional rates indicated an all causes standardized mortality ratio, SMR, lower than expected both in men (SMR = 0.91, 95%CI = 0.87-0.96) and women (SMR = 0.92, 95%CI = 0.87-0.96); cancer mortality and hospital admissions did not differ from the reference population, although some excesses in mortality were found for pleural cancer in men (SMR = 3.64, 95%CI = 1.66-6.91) and bladder cancer in women (SMR = 1.76, 95%CI = 1.01-2.86). Results for cancer incidence confirmed the pleural cancer excess in men (standardized incidence ratio, SIR = 2.14, 95%CI = 1.03-3.94), while an excess of breast cancer in women (SIR = 1.15, 95%CI = 1.03-1.27) was found.

Table 3 reports the results of the internal analyses of the association between heavy metal exposure and cause-specific mortality. No clear trend of all-cause or cause-specific mortality in relation to estimated heavy metals concentrations was observed in males. It should be noted, however, that an increase in mortality from all causes and from respiratory diseases was found in the second exposure category when compared with the reference. Also among women, no clear trend in all-cause and cause-specific mortality was observed. However, higher mortality was observed in all three exposure categories for all causes and for cardiovascular diseases when compared with the reference category.

**Table 3:** Associations between heavy metals concentration and mortality in the cohort of residents in the area of Coriano (Italy) by cause of death (ICD-9) and gender.

	**Heavy metals**^**†**^	Men				Women			
Cause (ICD-9)		**obs**^**§**^	**RR**^**§§**^	95% CI		**obs**^**§**^	**RR**^**§§**^	95% CI	
									
**All causes (0-999)**	**I**	549	1.00	-	-	495	1.00	-	-
	**II**	503	1.14*	1.00	1.29	514	1.19*	1.09	1.30
	**III**	502	1.05	0.92	1.19	460	1.09*	1.00	1.20
	**IV**	199	1.01	0.86	1.20	185	1.12*	1.00	1.27
									
**Cardiovascular diseases (390-459)**	**I**	215	1.00	-	-	195	1.00	-	-
	**II**	183	1.01	0.82	1.24	235	1.39*	1.14	1.70
	**III**	191	1.06	0.86	1.29	194	1.21	0.98	1.49
	**IV**	72	0.98	0.75	1.29	78	1.32	1.00	1.72
									
**Ischaemic heart diseases (410-414)**	**I**	101	1.00	-	-	73	1.00	-	-
	**II**	77	0.83	0.61	1.14	81	1.26	0.90	1.76
	**III**	77	0.93	0.68	1.26	75	1.24	0.88	1.73
	**IV**	27	0.79	0.51	1.22	25	1.14	0.72	1.82
									
**Respiratory diseases (460-519 )**	**I**	19	1.00	-	-	26	1.00	-	-
	**II**	31	2.07*	1.14	3.77	26	1.18	0.67	2.11
	**III**	23	1.35	0.72	2.53	19	0.92	0.50	1.70
	**IV**	7	1.01	0.42	2.45	4	0.53	0.18	1.56
									
**Chronic pulmonary -**	**I**	15	1.00	-	-	13	1.00	-	-
**diseases (490496)**	**II**	16	1.40	0.67	2.95	12	1.09	0.47	2.52
	**III**	14	0.99	0.47	2.12	10	0.93	0.40	2.19
	**IV**	3	0.53	0.15	1.86	1	0.27	0.03	2.06

* p-value < 0.05; † Categories of heavy metals: I (reference) <0.5 ng/m3; II 0.5-1 ng/m3; III 1-2 ng/m3; IV >2 ng/m3; § Observed cases; §§ Rate Ratios vs reference category, adjusted by age and socioeconomic status. Period: 1990-2003.

The results for hospital admissions for cardiovascular and respiratory causes (Table 4) in both sexes confirm the non-positive results observed for mortality. An increase in chronic heart failure only in men was found in the second category of exposure without a noteworthy trend.

**Table 4:** Associations between heavy metals concentration and hospitalization for specific causes in the cohort of residents in the area of Coriano (Italy) by cause (ICD-9) and gender.

	**Heavy metals**^**†**^	Men				Women			
Cause (ICD-9)		**obs**^**§**^	**RR**^**§§**^	95% CI		**obs**^**§**^	**RR**^**§§**^	95% CI	
**Acute Myocardic Infarction (AMI; 410)**^**‡**^	**I**	40	1.00	-	-	13	1.00	-	-
	**II**	29	0.76	0.44	1.30	15	1.08	0.48	2.41
	**III**	33	0.84	0.53	1.33	12	0.96	0.44	2.10
	**IV**	36	0.81	0.51	1.28	16	1.40	0.66	2.98
									
**Chronic heart failure (CHF; 428.0, 428.2, 428.9)**^**‡**^	**I**	30	1.00	-	-	27	1.00	-	-
	**II**	55	2.03*	1.25	3.29	24	1.04	0.58	1.87
	**III**	32	1.07	0.65	1.76	28	1.05	0.62	1.78
	**IV**	26	0.78	0.46	1.33	38	1.48	0.90	2.46
									
**Chronic obstructive pulmonary disease (COPD; 490-496; esc. 493)**^**‡**^	**I**	28	1.00	-	-	31	1.00	-	-
	**II**	39	1.46	0.85	2.50	24	0.69	0.38	1.27
	**III**	39	1.41	0.87	2.29	26	0.87	0.51	1.46
	**IV**	45	1.43	0.89	2.31	18	0.63	0.35	1.14
									
**Acute Respiratory Diseases (460-466; 480-487)**	**I**	67	1.00	-	-	70	1.00	-	-
	**II**	73	0.98	0.67	1.42	65	0.83	0.56	1.22
	**III**	80	1.18	0.86	1.64	63	0.91	0.65	1.28
	**IV**	63	0.89	0.63	1.27	90	1.29	0.94	1.78
									
**Asthma (493)**^**‡**^	**I**	6	1.00	-	-	10	1.00	-	-
	**II**	5	1.15	0.33	4.09	3	0.50	0.14	1.86
	**III**	1	0.19	0.02	1.58	5	0.59	0.20	1.74
	**IV**	6	1.16	0.36	3.71	9	1.01	0.40	2.55

* p-value < 0.05; † Categories of heavy metals: I (reference) <0.5 ng/m3; II 0.5-1 ng/m3; III 1-2 ng/m3 ; IV >2 ng/m3; § Observed cases; §§ Rate Ratios vs reference category, adjusted by age and socioeconomic status; ‡ Analyses for AMI, CHF and COPD was restricted to 35-74 year olds; analysis for asthma was restricted to 0-64 year olds. Period: 1999-2003

Table 5 reports the results for cancer mortality and cancer incidence for men and women. For men, no clear relation with increasing exposure to heavy metal was suggested for site-specific cancer mortality and incidence, with the only exception of colon-rectal cancer mortality that was doubled in the third and fourth exposure categories. On the contrary, a clear trend of increasing overall cancer mortality was seen among women. The all-cancer mortality results appeared mainly due to a gradient of increasing risk for stomach, colon, liver, breast, bladder and lympho-haemopoietic cancer (mainly non-Hodgkin's lymphoma and myeloma). Notably, the rate ratio for breast cancer in the highest exposure category was 2.00 (95%CI = 1.0-3.99). Cancer incidence data did not confirm the results found for mortality as no clear trend was detected.

**Table 5:** Associations between heavy metals concentration and mortality/incidence of cancer in the cohort of residents in the area of Coriano (Italy) by cause (ICD-9) and gender.

Cause (ICD-9)	**Heavy metals**^**†**^	Men								Women							
		mortality				cancer incidence				mortality				cancer incidence			
		**obs**^**§**^	**RR**^**§§**^	IC 95%		obs	**RR**^**†**^	IC 95%		**obs**^**§**^	**RR**^**§§**^	IC 95%		obs	**RR**^**§**^	IC 95%	
**All cancer (140-239)**	**I**	216	1.00	-	-	413	1.00	-	-	152	1.00	-	-	396	1.00	-	-
	**II**	194	1.12	0.92	1.38	327	0.94	0.81	1.08	154	1.24	0.98	1.57	323	0.96	0.83	1.11
	**III**	194	1.04	0.85	1.27	342	0.93	0.81	1.07	153	1.24	0.98	1.57	315	0.95	0.82	1.10
	**IV**	65	0.85	0.64	1.12	136	0.87	0.72	1.06	65	1.47*	1.09	1.99	112	0.90	0.73	1.11
																	
**Stomach (151)**	**I**	20	1.00	-	-	27	1.00	-	-	13	1.00	-	-	24	1.00	-	-
	**II**	20	1.44	0.76	2.73	28	1.18	0.69	2.00	13	1.32	0.59	2.98	22	1.02	0.57	1.81
	**III**	22	1.12	0.60	2.08	36	1.47	0.89	2.42	28	2.51*	1.27	4.97	31	1.54	0.91	2.63
	**IV**	7	0.85	0.35	2.03	13	1.24	0.64	2.40	7	1.86	0.73	4.75	8	1.09	0.49	2.44
																	
**Colon rectum (153-154)**	**I**	18	1.00	-	-	45	1.00	-	-	13	1.00	-	-	34	1.00	-	-
	**II**	11	0.61	0.28	1.35	31	0.82	0.52	1.29	16	1.32	0.61	2.87	27	0.91	0.55	1.51
	**III**	25	2.10*	1.10	4.00	51	1.28	0.86	1.91	19	1.94	0.93	4.06	56	2.00*	1.31	3.06
	**IV**	10	2.05	0.92	4.58	17	1.00	0.57	1.75	8	2.15	0.86	5.37	14	1.33	0.71	2.48
																	
**Liver (155)**	**I**	11	1.00	-	-	10	1.00	-	-	3	1.00	-	-	7	1.00	-	-
	**II**	6	0.61	0.21	1.75	7	0.80	0.30	2.10	3	0.92	0.17	5.11	2	0.30	0.06	1.46
	**III**	6	0.61	0.21	1.75	6	0.66	0.24	1.82	2	1.00	0.15	6.61	2	0.34	0.07	1.63
	**IV**	1	0.27	0.03	2.18	1	0.26	0.03	2.01	3	5.10	0.94	27.80	2	0.94	0.20	4.53
																	
**Larinx (161)**	**I**	6	1.00	-	-	18	1.00	-	-	0	1.00	-	-	2	1.00	-	-
	**II**	2	0.42	0.08	2.22	6	0.41	0.16	1.04	1	-	-	-	1	0.61	0.06	6.80
	**III**	3	0.53	0.13	2.25	4	0.26	0.09	0.76	1	-	-	-	1	0.60	0.05	6.62
	**IV**	0	0.00	0.00	.	1	0.15	0.02	1.14	0	-	-	-	1	1.60	0.15	17.64
																	
**Lung (162)**	**I**	54	1.00	-	-	69	1.00	-	-	15	1.00	-	-	16	1.00	-	-
	**II**	50	1.17	0.78	1.76	60	1.04	0.73	1.47	12	0.95	0.43	2.11	19	1.36	0.70	2.65
	**III**	56	1.15	0.78	1.71	64	1.05	0.75	1.48	10	0.89	0.39	2.06	11	0.82	0.38	1.78
	**IV**	18	0.91	0.53	1.57	25	0.96	0.61	1.52	4	0.96	0.31	2.97	4	0.81	0.27	2.42
																	
**Soft tissue sarcoma (171)**	**I**	0	1.00	-	-	3	1.00	-	-	0	1.00	-	-	1	1.00	-	-
	**II**	1	-	-	-	1	0.37	0.04	3.59	0	-	-	-	0	0.00	-	-
	**III**	0	-	-	-	2	0.72	0.12	4.31	2	-	-	-	4	4.71	0.53	42.16
	**IV**	1	-	-	-	1	0.84	0.09	8.06	2	-	-	-	0	0.00	-	-
																	
**Breast (175)**	**I**	0	1.00	-	-	0	1.00	-	-	21	1.00	-	-	125	1.00	-	-
	**II**	0	-	-	-	0	-	-	-	22	1.33	0.73	2.43	90	0.89	0.68	1.17
	**III**	0	-	-	-	0	-	-	-	18	1.02	0.55	1.92	81	0.78	0.59	1.03
	**IV**	0	-	-	-	0	-	-	-	13	2.00	1.00	3.99	30	0.76	0.51	1.13
																	
**Prostate (185)**	**I**	14	1.00	-	-	60	1.00	-	-	0	1.00	-	-	0	1.00	-	-
	**II**	12	1.08	0.50	2.33	48	0.93	0.64	1.37	0	-	-	-	0	-	-	-
	**III**	23	1.85	0.95	3.59	58	1.08	0.75	1.55	0	-	-	-	0	-	-	-
	**IV**	8	1.57	0.66	3.74	29	1.27	0.82	1.99	0	-	-	-	0	-	-	-
																	
**Bladder (188)§**	**I**	10	1.00	-	-	48	1.00	-	-	4	1.00	-	-	7	1.00	-	-
	**II**	13	1.50	0.62	3.61	33	0.83	0.53	1.29	4	1.09	0.25	4.78	9	1.49	0.55	4.01
	**III**	14	1.50	0.64	3.51	32	0.76	0.48	1.18	3	1.00	0.21	4.82	5	0.85	0.27	2.68
	**IV**	6	1.48	0.52	4.22	14	0.78	0.43	1.42	3	3.06	0.64	14.70	5	2.30	0.73	7.24
																	
**Central nerv. sys. (191-192;225)§**	**I**	4	1.00	-	-	6	1.00	-	-	4	1.00	-	-	8	1.00	-	-
	**II**	3	0.51	0.10	2.55	9	1.84	0.65	5.17	4	0.76	0.17	3.43	5	0.77	0.25	2.36
	**III**	5	1.65	0.38	7.21	7	1.32	0.44	3.93	6	2.38	0.61	9.21	6	0.90	0.31	2.61
	**IV**	0	0.00	-	-	3	1.35	0.34	5.39	0	0.00	-	-	0	0.00	-	-
																	
**Lymphoemat.system (200-208)**	**I**	27	1.00	-	-	50	1.00	-	-	17	1.00	-	-	34	1.00	-	-
	**II**	19	0.87	0.47	1.62	31	0.75	0.48	1.18	14	0.93	0.44	1.97	29	1.02	0.62	1.67
	**III**	14	0.58	0.30	1.14	34	0.77	0.50	1.19	12	0.94	0.44	2.05	23	0.81	0.48	1.38
	**IV**	4	0.42	0.15	1.23	13	0.70	0.38	1.28	8	1.78	0.74	4.25	13	1.23	0.65	2.33
																	
**Non-Hodgkin Limphoma (200,202)**	**I**	10	1.00	-	-	23	1.00	-	-	7	1.00	-	-	15	1.00	-	-
	**II**	6	0.80	0.28	2.29	10	0.54	0.26	1.14	7	0.83	0.27	2.56	14	1.10	0.53	2.29
	**III**	6	0.63	0.22	1.82	13	0.65	0.33	1.28	2	0.47	0.09	2.44	8	0.64	0.27	1.51
	**IV**	2	0.52	0.11	2.45	5	0.59	0.23	1.57	3	2.03	0.48	8.67	5	1.06	0.39	2.93
																	
**Myeloma (203)**	**I**	7	1.00	-	-	13	1.00	-	-	3	1.00	-	-	8	1.00	-	-
	**II**	3	0.37	0.09	1.62	14	0.45	0.16	1.27	1	0.32	0.03	3.53	9	0.36	0.10	1.30
	**III**	2	0.33	0.06	1.72	11	0.61	0.24	1.52	3	1.44	0.26	7.93	9	0.48	0.15	1.54
	**IV**	0	0.00	-	-	5	0.61	0.17	2.13	3	4.28	0.77	23.80	3	0.95	0.26	3.45
																	
**Leukaemia (204-208)**	**I**	9	1.00	-	-	13	1.00	-	-	5	1.00	-	-	10	1.00	-	-
	**II**	9	1.33	0.51	3.49	5	1.27	0.60	2.72	6	1.82	0.54	6.19	3	1.31	0.50	3.40
	**III**	6	0.78	0.27	2.25	7	0.94	0.42	2.11	7	1.69	0.52	5.55	4	1.35	0.52	3.49
	**IV**	2	0.67	0.14	3.16	3	1.01	0.36	2.84	2	1.31	0.25	6.95	3	1.23	0.33	4.62

* p-value < 0.05; ^† ^Categories of heavy metals: I (reference) <0.5 ng/m^3^; II 0.5-1 ng/m^3^; III 1-2 ng/m^3 ^; IV >2 ng/m^3^; § Observed cases; §§ Rate Ratios (RR) versus the reference category of heavy metals adjusted for age and socioeconomic status; ‡ ICD-9 codes considered for cancer incidence were: bladder cancer = 188;223.3;223.7;236.7;239.4; cancer of the central nervous system = 191-192. Period: 1990-2003.

No cases were observed in the reference category for soft tissue sarcoma, therefore rate ratios could not be calculated. On the other hand, four incident cases were found in the third category of heavy metals among women (RR = 4.71, not statistically significant, n.s.). When results for men and women were combined, six deceased cases were found and an excess in the highest category of exposure (RR = 16.54; 95%CI = 1.72-159.07) was detected.

When all analyses were repeated considering exposure to NO_2 _as a potential confounder, none of the rate ratios estimates changed substantially and the overall findings were confirmed (data not shown).

We performed an additional analysis of mortality considering only residents who were present in the study area in 1990. This sub-cohort represents the 78.8% of the total cohort (91.3% of the total person-years). The results were substantially similar with wider confidence intervals although statistical significance was reached for liver cancer among women at the 4th level of exposure (RR = 7.39; 95%CI = 1.11-49.10, based on 10 cases).

## Discussion

We evaluated mortality, cancer incidence and hospitalization for cardiovascular and respiratory diseases among people living close to incinerators using dispersion modelling to assess exposure. The internal analyses showed no association with non-cancer related mortality and morbidity. However, predicted heavy metals concentrations, as indicator of air pollution from the incinerators, were somehow related to cancer mortality in women, in particular for stomach, colon, liver and breast cancer. In addition, a combined analysis of men and women suggested an increase in soft-tissue sarcoma mortality related to exposure to incinerators. The results were adjusted for socioeconomic status whereas there was no important confounding effect from pollution due to other sources.

The excesses detected in the areas with higher exposure levels were observed mainly among females. Of course, a chance finding could be an explanation given multiple testing but it should also considered that women are a more stable population than men and misclassification of exposure is less likely to have occurred. On the other hand, most of the associations that were found for mortality cancer outcomes were not confirmed by incidence data although the time window of follow-up was the same (1990-2003). A possible explanation of these findings is that the effect of the exposure on cancer incidence precedes the time window of our study so that only mortality is affected.

From the results of the present study it is difficult to determine the causality of the associations and which specific agent emitted from the plants could have an etiological role in the excess risk that we have found. Of course, we considered heavy metals as a surrogate marker for exposure to a complex mixture of pollutants. As for the results of other studies on incinerators, the role of exposure to dioxins could be of importance in this context. Dioxin refers to 210 congeners/isomers of structurally and chemically related polychlorinated *dibenzo*-*para*-*dioxins *(PCDDs) and polychlorinated *dibenzofurans *(PCDFs), and the 2,3,7,8-tetra-CDD (TCDD) is considered the most toxic dioxin congener in this group. Dioxins are persistent in the environment and resistant to biodegradation and are considered human carcinogens [21]. The MSW incinerator in Forlì did emit dioxins and the values were considered relatively high until 1996 [16]. Like in our female population, increases for all cancers and in particular for cancer of the digestive system (stomach and colon rectum) have been observed among occupational cohorts exposed to dioxin [22] and in the Seveso population, among residents in the more contaminated areas [23]. In addition, among women we observed an increase in mortality for Hodgkin's disease and myeloma (based on few cases and not statistically significant) as it has been reported among Seveso women [24] and in a French study [11] where the increase of blood cancer was related to dioxin exposure from incinerators. We observed a clear excess for breast cancer but the literature on the risk of breast cancer in the proximity of incinerators is rather poor [1,2]. No breast cancer excesses were observed in Seveso's longitudinal study, following the 1976 accident [25]; however, the Seveso Women's Health Study reported a two-fold risk for breast cancer among pre-menopausal women with highest serum levels of TCDD [26]. There are several other studies that have found increased breast cancer incidence [27-29] in females occupationally exposed to dioxins. Finally, as in our observation, several studies have related residency in proximity of incinerators with liver cancer [8] and soft tissue sarcoma [10,2,14,30,31] although negative results also exist [32].

The strength of this work is the longitudinal study design adopted, in which individuals were followed for various health outcomes, exposure was assessed with advanced modelling techniques, socioeconomic status and other environmental exposures were also considered as potential confounders. To our knowledge, there are no other studies on incinerators conducted at the individual level with the details that we took into account. Despite that, the methodological aspects of the study and the main limitations should be considered.

Exposure assessment is a critical component of the study. We define the study population as people living up to 3.5 km far from the plants. This choice is based on previous studies [8,10] and information by model simulations on the profile of the distribution of pollutant emitted by plants. In fact, this choice provided a good contrast of exposure conditions and a better comparability of the contrasted population groups. We geo-coded all the residential addresses and exposure was assessed using the results of a model of dispersion of pollutants into the atmosphere. A French study has validated Gaussian dispersion model for dioxins from an incinerator with a campaign of measurements on the ground in 75 sampling points [33]. The results confirmed the validity of the model in defining the different gradients of exposure, and identified inconsistencies between measured levels and those estimated by the model only in the presence of complex topographical situations (e.g. hills), a condition that does not apply to the Po Valley. A recent British study compared the use of distance as a proxy of exposure from a source of pollution by means of estimates derived from dispersion models and concluded that the use of the models significantly reduces the risk of misclassification embedded in the use of the distance from a point source [34].

The approach we used for exposure assessment has several assumptions and limitations. First of all, we considered only exposure to air pollution whereas other exposure routes, such as soil contamination or food and water consumption, could have importance. We considered only the individual residences at the beginning of the follow-up and this choice was supported by the observation that the exposure category never changed during the study for over 90% of the subjects (Table 2). Also, exposure was assessed at the beginning of follow-up to account for diseases with a long period of induction-latency (such as cancer) where the relevant exposure does not necessarily correspond to when it was diagnosed but to exposure levels in the previous years or decades. We used authorized emission values of pollutants to simulate dispersion from incinerators. This could have overestimated concentration values, but the shape of the fallout and the gradients of exposure are not sensitive to this choice. Finally, we assumed that heavy metals are better tracer for incinerator pollution than other pollutants since there is vast literature that indicates different heavy metals (such as Cd, Ni, As, Pb, Zn, Cu, Mn) as possible tracers of incinerators [16,35,36], and this choice was supported by monitoring campaigns conducted during the study period.

The role of the potential confounders, in particular other occupational and/or environmental exposures, should be considered. We observed a cluster of incidence and mortality for pleural cancer among men in the second exposure category; the absolute number of cases is low, but the relative risk is high. This cluster is due to occupational exposure to asbestos in the small industrial area in the second exposure category. There are other environmental factors in the study area due to the proximity of the highway and the urban area. We took into account the effect of exposure to vehicular traffic using predicted NO_2 _levels; the dispersion model for NO_2 _showed no overlap between the areas of high NO_2 _and the areas of high heavy metal levels. The traffic-related pollutants in this case would act, at least in theory, as negative confounders. However, when NO_2 _levels where considered in the analysis no important changes in the relative risk estimates were noted, even for respiratory mortality.

An additional limit, like many other epidemiological investigations, is the lack of individual data about potential confounding factors such as individual socioeconomic conditions, occupational exposure, and personal lifestyle factors such as smoking habits. Data on socioeconomic status, available at the aggregate level (census tract), allowed us to indirectly take into account other factors related to mortality and/or cancer incidence (i.e. smoking habits and occupational exposure are strongly linked to socioeconomic conditions). In fact, rate ratios estimates were attenuated after adjusting for socioeconomic status (all causes, cardiovascular diseases and some cancer types; data before adjustment are not reported). On the basis of these findings, we cannot exclude the possibility of a residual confounding by socioeconomic status.

It is notable that the lack of information about residential history before 1990 has limited the possibility of evaluating the effects of duration of exposure and latency since first exposure, two common useful measures in cohort analysis. Finally, as already indicated, power limitations given the size of the population studied may have limited the possibility to provide stable results. In fact, the study was able to detect a relative risk of 1.3 in the last category of exposure for all cancer combined (with α = 0.95 and β = 0.80).

## Conclusions

We found some excesses of cancer mortality among residents in areas with the highest predicted concentration of heavy metals. These findings might be possibly related to pollutants released from the incinerators over the past decades. This study contributes to the controversy over the possible health effects of waste management. However, future research into the health risks of waste management needs an accurate characterization of individual exposure, an improved knowledge of chemical and toxicological data of specific compounds, multi-site studies of large populations to increase statistical power, approaches based on individuals rather than communities and a better control of confounding factors. In this view, an ongoing multi-site project over the entire Emilia-Romagna region is investigating possible health effects due to exposure to all eight incinerators operating in the region.

## List of Abbreviations

ADMS: Atmospheric Dispersion Model System; ARPA: Regional Agency for Environmental Prevention; As: Arsenic; BTX: Benzene, Toluene, Xilene; C6H6: Benzene; Cd: Cadmium; CO: Carbon monoxide; Cu: Copper; GIS: Geographic Information System; HCl: Hydrochloric acid; HW: Hospital Waste; Mn: Manganese; MSW: Municipal Solid Waste; Ni: Nickel; NO2: nitrogen dioxide; PAH: Polycyclic aromatic hydrocarbon; Pb: Lead; PCB: Polychlorinated biphenyls; PCDD: Polychlorinated dibenzodioxins; PCDF: Polychlorinated dibenzofurans; RR: Rate Ratio; SD: Standard Deviation; SES: Socioeconomic Status; SIR: Standardized Incidence Ratio; SMR: Standardized Mortality Ratio; SOx: Sulfur Oxides; TCDD: 2,3,7,8-Tetrachlorodibenzo-p-dioxins; TSP: Total Suspended Particles; VOC: Volatile Organic Compounds; Zn: Zinc.

## Competing interests

The authors declare that they have no competing interests.

## Authors' contributions

AR and VF participated in the design of the study, performed the statistical analysis and drafted the manuscript. LE participated in the acquisition of environmental data and exposure assessment. PL participated in study conceiving and design. CAP has been involved in coordination and interpretation. FF coordinated the design of the study, the statistical analyses and helped to draft the manuscript. All authors read and approved the final manuscript.
